# Moths passing in the night: Phenological and genomic divergences within a forest pest complex

**DOI:** 10.1111/eva.13338

**Published:** 2022-01-11

**Authors:** Tyler D. Nelson, Zachary G. MacDonald, Felix A. H. Sperling

**Affiliations:** ^1^ Department of Biological Sciences University of Alberta Edmonton Alberta Canada; ^2^ Department of Renewable Resources University of Alberta Edmonton Alberta Canada; ^3^ Summerland Research and Development Centre Agriculture and Agri‐Food Canada Summerland British Columbia Canada

**Keywords:** allochrony, ecological speciation, periodicity, two‐year cycle spruce budworm

## Abstract

Temporal separation of reproductive timing can contribute to species diversification both through allochronic speciation and later reinforcement of species boundaries. Such phenological differences are an enigmatic component of evolutionary divergence between two major forest defoliator species of the spruce budworm complex: *Choristoneura fumiferana* and *C. occidentalis*. While these species interbreed freely in laboratory settings, natural hybridization rates have not been reliably quantified due to their indistinguishable morphology. To assess whether temporal isolation is contributing to reproductive isolation, we collected adult individuals throughout their expected zone of sympatry in western Canada at 10‐day intervals over two successive years, assigning taxonomic identities using thousands of single nucleotide polymorphisms. We found unexpectedly broad sympatry between *C. fumiferana* and *C. occidentalis biennis* and substantial overlap of regional flight periods. However, flight period divergence was much more apparent on a location‐by‐location basis, highlighting the importance of considering spatial scale in these analyses. Phenological comparisons were further complicated by the biennial life cycle of *C. o. biennis*, the main subspecies of *C. occidentalis* in the region, and the occasional occurrence of the annually breeding subspecies *C. o. occidentalis*. Nonetheless, we demonstrate that biennialism is not a likely contributor to reproductive isolation within the species complex. Overall, interspecific F_1_ hybrids comprised 2.9% of sequenced individuals, confirming the genomic distinctiveness of *C. fumiferana* and *C. occidentalis*, while also showing incomplete reproductive isolation of lineages. Finally, we used *F*_ST_‐based outlier and genotype–environment association analyses to identify several genomic regions under putative divergent selection. These regions were disproportionately located on the Z linkage region of *C. fumiferana*, and contained genes, particularly antifreeze proteins, that are likely to be associated with overwintering success and diapause. In addition to temporal isolation, we conclude that other mechanisms, including ecologically mediated selection, are contributing to evolutionary divergence within the spruce budworm species complex.

## 1 INTRODUCTION

Temporal isolation of potentially interbreeding evolutionary lineages can be a significant contributor to species diversification (Hendry & Day, 2005; Nosil, 2012; Rundle & Nosil, 2005). Such phenological divergences may occur at different temporal scales, whether within a day (e.g., Devries et al., 2008; Schöfl et al., 2009; Ueno et al., 2006), seasonally (e.g., Adamski et al., 2018; Bell et al., 2017; Yamamoto & Sota, 2009), or between years (Cooley et al., 2003; Gradish et al., 2015). Multiple ecological and physiological processes may lead to temporal isolation, and it is almost always difficult to determine whether present‐day phenological divergence was the initial cause or a later reinforcing consequence of reproductive isolation (Egan et al., 2015; Taylor & Friesen, 2017). In any case, the essential first step to inferring whether phenological divergences are contributing to contemporary reproductive isolation among taxa is to rigorously document the extent and nature of their temporal separation and associated characteristics.

**FIGURE 1:**
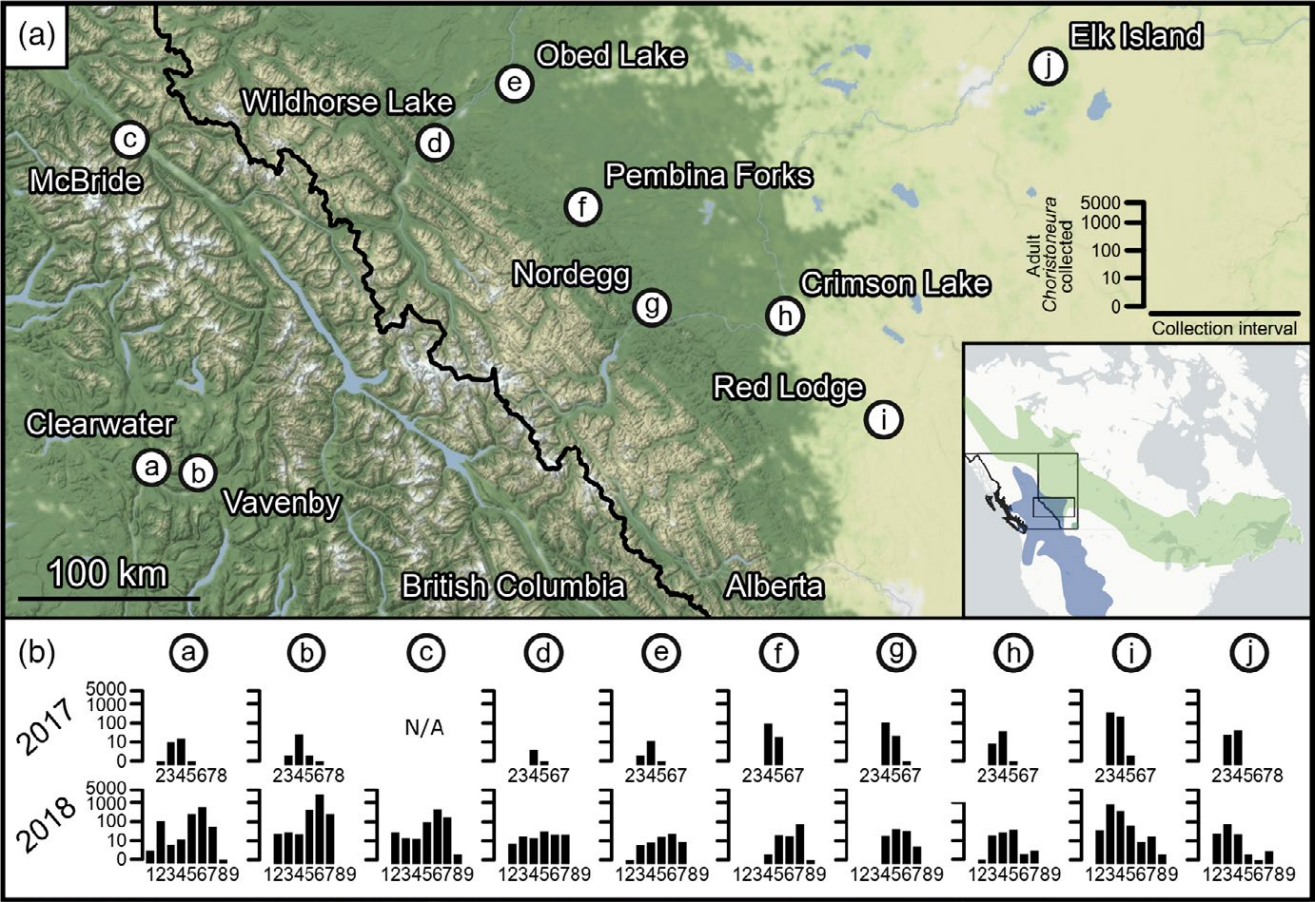
Collection locations (a) and phenology (b) of adult *Choristoneura* across the study area. Moths were pheromone‐trapped at each location during ~10 days before collection. Histograms show numbers of adult *Choristoneura* trapped in each collection interval, with traps being emptied on: 1 = 11–15 June, 2 = 21–27 June, 3 = 1–7 July, 4 = 11–17 July, 5 = 21–27 July, 6 = 31 July ‐ 6 August, 7 = 10–17 August, 8 = 20–24 August, 9 = 30 August ‐ 2 September (Tables S2 and S3 show days since 1 January for each location). Collection intervals without number digits from 2017 indicate traps were not deployed, while numbered collection intervals without histogram bars indicate no moths were trapped. Inset map of North America shows rectangular study area in Alberta and British Columbia with *C. fumiferana* (green) and *C. occidentalis* (blue) ranges (adapted from Dupuis et al., 2017). Map was prepared in QGIS version 3.6.0 using base maps obtained from https://github.com/stamen/terrain‐classic and https://github.com/CartoDB/basemap‐styles

The economically important North American spruce budworm species complex contains two spruce/fir‐feeding species, *Choristoneura fumiferana* (Clemens) and *C. occidentalis* Freeman, that exhibit very similar morphology (French et al., 2020; Lumley & Sperling, 2010), sex pheromones (Silk & Eveleigh, 2016), and life‐history traits (Brunet et al., 2013; Volney & Fleming, 2007). *Choristoneura fumiferana* ranges across the northeastern United States and much of Canada's boreal forest, feeding mainly on white spruce (*Picea glauca* (Moench) Voss) and balsam fir (*Abies balsamea* (L.) Mill.) (Lumley & Sperling, 2011b) (Figure 1). *Choristoneura occidentalis* occurs in western North America, where larvae feed mainly on Douglas fir (*Pseudotsuga menziesii* (Mirb.) Franco) as the subspecies *C. o. occidentalis*, and on subalpine fir (*Abies lasiocarpa* (Hooker) Nuttall) and Engelmann spruce (*Picea engelmannii* Parry ex Engelm.) as *C. o. biennis* Freeman (Brunet et al., 2017). While *C. o. occidentalis* and *C. o. biennis* are parapatric in British Columbia and Alberta, Canada, each shares a substantial zone of sympatry with *C. fumiferana* in western Alberta (Dupuis et al., 2017; Lumley & Sperling, 2011a, 2011b). Intriguingly, pairings of *C. fumiferana* and *C. occidentalis* hybridize successfully in laboratory settings (Harvey, 1997; Nealis, 2005; Sanders et al., 1977), but few genetically admixed individuals indicative of hybridization have been observed in nature (Brunet et al., 2017; Lumley & Sperling, 2011a). This suggests that *C. fumiferana* and *C. occidentalis* are reproductively isolated, but specific mechanisms that underlie their isolation are not well understood.

Seasonal flight peaks of *C. fumiferana* and *C. occidentalis* have been inferred to differ within their zone of sympatry in Alberta (Lumley & Sperling, 2011a). Assuming flight peaks are adequate proxies of reproductive timing for each species, divergence of flight peaks suggests that temporal isolation may be contributing to their continued reinforcement as distinct species (Brunet et al., 2017). However, although flight peaks may differ between *C. fumiferana* and *C. occidentalis*, any degree of flight period overlap would suggest that gene flow among these sympatric species is possible, barring other pre‐ or post‐zygotic reproductive incompatibilities. Therefore, explicit quantification of flight period overlap is needed and requires surveys repeated at regular intervals spanning the zone of *C. fumiferana*/*C. occidentalis* sympatry. Another important consideration is that assigning species identifications to *Choristoneura* individuals is generally unreliable without genomic data (French et al., 2020), weakening inferences of phenological divergence based on historical data, morphology, sex pheromones, or life‐history traits (e.g., Liebhold & Volney, 1984; Powell & De Benedictus, 1995; Smith, 1954; Volney et al., 1984). Targeted phenological studies using genomic data are therefore required to resolve mechanisms that contribute reproductive isolation within this species complex.

In addition to seasonal divergences in the flight periods of *C. fumiferana* and *C. occidentalis*, it has also been hypothesized that biennialism contributes to their reproductive isolation (Shepherd et al., 1995). While *C. fumiferana* and *C. o. occidentalis* are univoltine, larvae of *C. o*. *biennis* undergo an obligate second diapause and overwinter a second time before completing a two‐year (semivoltine) life cycle (Harvey, 1967; Nealis, 2005). This biennialism results in adult *C. o*. *biennis* flying in high abundance every second year, separated by a year of very few adults (Nealis & Turnquist, 2003; Shepherd et al., 1995; Zhang & Alfaro, 2002). However, biennialism on its own cannot account for complete reproductive isolation among sympatric taxa. If sex ratios, abundances, and flight periods of two sympatric taxa are very similar and they do not exhibit prezygotic barriers to reproduction, interspecific mating will not occur in years in which only one taxon reproduces but will be substantial for years in which both taxa reproduce. In other words, reproductive isolation would not be maintained in years in which both semivoltine *C. o. biennis* and univoltine *C. fumiferana* emerge as adults. Nonetheless, variation in voltinism may still indirectly contribute to within‐year reproductive isolation if *C. o. biennis* flies earlier than *C. fumiferana* due to a phenological “head start” in years when both emerge as adults. This hypothesis would be supported if *C. o. biennis* populations consistently fly before sympatric *C. fumiferana* populations, but must be tested at fine temporal and spatial grains using genomic data to consistently assign taxon identities to individuals.

In this study, our principal aims were to (1) determine whether the three closely related spruce budworm taxa, *C. fumiferana*, *C. o. occidentalis*, and *C. o. biennis*, are temporally isolated in their zone of sympatry in west‐central Alberta, Canada; (2) quantify the extent of natural hybridization between these taxa; and (3) identify whether there are particular genomic regions under putative divergent selection within the *C. fumiferana* species complex, indicating ecologically mediated divergent selection. *Choristoneura fumiferana*, *C. o. occidentalis*, and *C. o. biennis* were collected across their zone of sympatry and surrounding areas over two successive years and genotyped using next‐generation sequencing to assign taxon identities to sampled individuals. We also identified genomic regions under putative divergent and ecologically mediated selection, using a combination of *F*_ST_ outlier and genotype–environment association analyses, to identify additional factors that may be contributing to genomic variation and reproductive isolation among these taxa.

## 2 METHODS

### 2.1 Specimen collection

We sampled adult *Choristoneura* at 10 locations in Alberta and British Columbia at 10‐day intervals during their 2017 and 2018 flight seasons (Figure 1, Table S1). Six collection locations in Alberta (d–i, Figure 1) were within the documented zone of sympatry of *C*. *fumiferana* and *C. o*. *biennis* (Brunet et al., 2017). Three locations were chosen in British Columbia (a–c) outside of the known range of *C. fumiferana* (Dupuis et al., 2017) based on aerial surveys depicting severe *C. o. biennis* defoliation in 2015 and 2016 (Westfall & Ebata, 2016, 2017). The 10^th^ location (Elk Island, Alberta) was geographically distant from the documented range of *C. o. biennis* and therefore expected to contain only *C. fumiferana* (Brunet et al., 2017; Lumley & Sperling, 2011b).

Adult moths were collected during 13 June—25 August 2017 and 1 June—3 September 2018 (Tables S2 and S3). At each of the 10 locations, four green Unitraps (Contech, Delta, British Columbia, Canada) were hung two meters above ground level on mature white spruce, Engelmann spruce, or Douglas fir, with at least 40 meters between each trap to reduce interference (Allen et al., 1986; Sanders, 1996). Three Unitraps were baited with rubber septum lures containing 100 μg of synthetic 95:5 *E*,*Z*‐11‐tetradecanal (Sylvar Technologies, Fredericton, New Brunswick, Canada), the dominant component of the *C. fumiferana* sex pheromone (Silk & Kuenen, 1988). The 95:5 *E*,*Z* blend developed for male *C. fumiferana* attraction effectively attracts other male *Choristoneura*, including *C. o*. *biennis* and *C. o. occidentalis* (Sanders et al., 1977; e.g., Lumley & Sperling, 2011a, 2011b; Brunet et al., 2017), although the optimum *E*/*Z* ratio for *C. o. biennis* is unknown (Sanders, 1971; Sanders et al., 1974). The fourth trap did not contain a lure, acting as a control. We included one strip of 10% dichlorvos in each trap as a killing agent (Hercon Environmental, Emigsville, Pennsylvania, USA). Trap catch was collected every 10 days and specimens were then stored at −20°C. When we began our study in 2017, aerial reports from 2015 and 2016 did not resolve whether even‐ or odd‐year defoliation was dominant, so we could not be sure which year *C. o. biennis* was likely to fly in high abundances (Westfall & Ebata, 2016, 2017). We therefore continued surveys in 2018, adding McBride (location c) based on historical records of severe even‐year defoliation by larval *C. o. biennis* in the area (Westfall & Ebata, 2017).

### 2.2 DNA extraction and ddRADseq

A minimum of three adult individuals were randomly subsampled for genotyping within each collection period at each collection location when available. Up to 10 individuals were genotyped from collection intervals if samples were numerous. To maximize coverage of total flight period, we also selected singletons that were trapped earliest or latest in each collection season. Whenever possible, we selected both fresh and worn moths to cover the breadth of variability present. Individuals that were wet upon collection were not selected for genotyping due to the possibility of degraded genomic DNA.

DNA was extracted using DNeasy Blood & Tissue DNA Kits (QIAGEN, Hilden, Germany), with the addition of bovine pancreatic ribonuclease A treatment (RNaseA, 4 μl at 100 mg/ml; Sigma‐Aldrich Canada Co., Canada). DNA of each specimen was ethanol precipitated and resuspended in millipore water for further concentration and purification, then stored at −20°C until library preparation. *PstI*‐*MspI* restriction enzyme ddRADseq libraries were prepared according to the protocol outlined in MacDonald et al. (2020). Single‐end, 75 base‐pair (bp) sequencing was performed on an Illumina NextSeq 500 platform. Sequencing of all individuals required three separate runs. Four individuals were sequenced twice to assess run effects. Remaining wings, head, and abdomen tissue from sequenced individuals are retained in the E. H. Strickland Entomological Museum for future reference and verification.

### 2.3 DNA data processing

Single‐end, 75 bp Illumina reads were demultiplexed using the *process_radtags* program in Stacks 2 version 2.3e (Rochette et al., 2019). We discarded any reads with Phred scores <20 over 15% of the read length and any reads that failed the Illumina purity filter. Retained reads were trimmed from 75 bp to 67 bp after removing the 8 bp index sequence. Some sequencing error was identified in the *PstI* site, so another 5 bp were removed from the 5’ end with Cutadapt version 1.18 (Martin, 2011), resulting in a final read length of 62 bp.

We aligned these 62 bp reads to a 30,339 scaffold *C. fumiferana* draft genome, bw6 (Larroque et al., 2019), using Burrows‐Wheeler Aligner version 0.7.17‐r1188 (Li & Durbin, 2009). Resulting SAM files were converted to BAM format for use in the Stacks 2 pipeline with the *ref_map* program stipulating a single population containing all individuals (Rochette et al., 2019). Stacks‐filtered single nucleotide polymorphisms (SNPs) were further filtered in VCFtools version 0.1.16, using missing data across SNPs and minor allele frequency thresholds of 95% and 0.10, respectively (Danecek et al., 2011). We also thinned SNPs that were within 10k bp of one another, to minimize physical linkage (MacDonald et al., 2020).

### 2.4 Taxonomic identifications and hybrid individuals

Taxon identities were determined with a combination of principal component analysis (PCA), using the R package *adegenet* version 2.1.1 (Jombart & Ahmed, 2011), and model‐based clustering analyses, using the programs *structure* version 2.3.4 (Falush et al., 2003; Pritchard et al., 2000) and NewHybrids version 1.1 beta3 (Anderson & Thompson, 2002). For *structure* analyses, we ran 10 iterations for each of *k* = 1–10 with a burn‐in period of 100,000 followed by 1,000,000 Markov chain Monte Carlo repetitions. The *locpriors* parameter, with values corresponding to the 10 collection locations, was used to better resolve spatial genetic structure without biasing the model (Porras‐Hurtado et al., 2013). We used StructureSelector (Li & Liu, 2018) to determine the number of genetic clusters with the highest support using both LnP(*k*) (Pritchard et al., 2000) and Δ*k* (Evanno et al., 2005). CLUMPAK version 1.1 (Kopelman et al., 2015) was used to average the 10 replicates of each *k* and create matrices of admixture coefficients (Jakobsson & Rosenberg, 2007). For NewHybrids analysis, we averaged the results of 5 iterations of the *k* = 2 dataset. Each iteration ran with a burn‐in period of 100,000 followed by 1,000,000 sweeps. Because NewHybrids experiences underflow errors with large datasets (Elliot & Russello, 2018; Hu et al., 2021), we chose the first 400 SNPs in the dataset for easier reproducibility (Gramlich et al., 2018). We also ran a second analysis using 400 randomly chosen SNPs to check if results were congruent (Gramlich et al., 2018). In both NewHybrids analyses, we assigned individuals to the two parental classes (parental 1, parental 2) using >99% membership to the two genetic clusters found by *structure* (Bell et al., 2015).

Individuals were identified as either *C. fumiferana* or *C. occidentalis* (*C. o. biennis* + *C. o. occidentalis*) if they had >90% membership to either genetic cluster based on *structure* admixture coefficients for *k* = 2. We used NewHybrids to assign hybrid class membership to each individual (parental 1, parental 2, F_1_, F_2_, backcross to parental 1, and backcross to parental 2), executed in EasyParallel version 1.0 (Zhao et al., 2020). We then reran *structure* to resolve substructure within each of the two species (primary genetic clusters). F_1_ hybrids, as determined by NewHybrids, were excluded in the second runs. VCFtools was used to subset the genomic dataset for each species and refilter SNPs in accordance with the filtering parameters detailed above. Relationship between *C. o. biennis* and *C. o. occidentalis* was inferred based on admixture coefficients >50% (Blackburn et al., 2017; Brunet et al., 2017).

### 2.5 Evaluation of temporal isolation

We estimated temporal overlap of *C. fumiferana* and *C. occidentalis* flight periods using the taxonomic identities of sequenced individuals and two co‐occurrence indices. The first index was calculated as the number of collection periods in which both *C. fumiferana* and *C. occidentalis* were present divided by the total collection periods that either species was present. This was first completed on a location‐by‐location basis for all sympatric collection locations. Resulting values were averaged across these locations to give an overall measure of temporal overlap. We also calculated this index by first pooling all individuals from all sympatric collection locations and then dividing the number of collection periods in which both species were present by the total collection periods that either species was present. Comparison of index values calculated on a location‐by‐location vs. pooled‐location basis allowed us to infer whether combining species occurrence records across a broad spatial extent overestimates the actual temporal overlap of sympatric populations. We also used a second index using species’ abundances. This index was calculated as the total number of *C. fumiferana* and *C. occidentalis* individuals that were collected in the same, pooled‐location, collection interval divided by the total number sequenced individuals across all collection intervals at all locations. This was again completed on a location‐by‐location basis. F_1_ hybrids were not included in either index.

To allow more controlled comparisons among locations under varied climatic conditions, we related phenology to local thermal accumulation using degree‐day estimations (Zalom et al., 1983). We recorded degree‐day accumulation using HOBO Pendant Temperature/Light Data Loggers (Onset Computer Corporation, Bourne, Massachusetts, USA) during the 2018 collection season, with data loggers hung two meters above ground in the shade at each location. Resulting temperature data were then calibrated against records for nearest weather stations (Table S4). When no stations were within 15 km of a location, we compared multiple nearby stations. Data downloaded from all selected stations were used to estimate highest and lowest temperatures for all days between trap set‐up and removal for both 2017 and 2018. Wilcoxon–Mann–Whitney tests were used to evaluate differences between logger and station temperatures (Table S5). We applied median difference between logger and station temperature as a correction factor applied to all daily high and low temperatures recorded by weather stations between 1 January and trap removal. Régnière (1990) showed that photoperiod has minimal effect on postdiapause emergence of *C*. *fumiferana*, so we did not use this index in our analyses.

We used degree‐day accumulation to compare phenology between disparate locations. This was calculated using single triangulation (Lindsey & Newman, 1956; Zalom et al., 1983), which is more accurate than alternatives when temperatures are far below the lower developmental threshold of an insect (Roltsch et al., 1999). We used 5.5°C as the lower development threshold for both *C. fumiferana* and *C. o. biennis* based on previous work (e.g., Kemp et al., 1986; Shepherd, 1961). We did not use an upper developmental threshold, as temperatures only exceeded the *C. fumiferana* threshold of 38°C (Régnière et al., 2012) during one‐hour‐long period at Red Lodge (location i). To summarize results, we plotted the number of genotyped individuals collected each 10‐day interval against the number of within‐location accumulated degree‐days for each location.

### 2.6 Genomic regions with high divergence

We used BayeScan version 2.1 (Foll & Gaggiotti, 2006) to identify specific genomic regions that were highly diverged among *Choristoneura* taxa. BayeScan has been recognized as an effective method for identification of *F*_ST_ outlier loci between discrete genetic clusters that may warrant further investigation using whole‐genome sequence data (De Mita et al., 2013; Lotterhos & Whitlock, 2014; Narum & Hess, 2011). *F*_ST_ values were estimated for all variable loci shared between *C. fumiferana* and *C. occidentalis* (*C. o. biennis* + *C*. *o. occidentalis*) and between *C. o. biennis* and *C. o*. *occidentalis*. Assignment of individuals to either *C*. *fumiferana* or *C. occidentalis* was based on admixture coefficients greater than 0.9 in *structure* analyses. We assigned *C. occidentalis* subspecies identities using admixture coefficients greater or less than 0.5. For all Bayescan runs, prior odds were set to 10, thinning interval to 10, number of pilot runs to 20, length of pilot runs to 5000, burn‐in length to 50,000, and number of output iterations to 10,000. We assessed significance of *F*_ST_ outliers using the false discovery rate (FDR) criterion and a *q*‐value threshold of 0.05 (−log10 q‐value ~ 1.3) (Benjamini & Hochberg, 1995). Fifteen BayeScan runs were completed for both *C. fumiferana* vs. *C. occidentalis* and *C. o. biennis* vs. *C. o*. *occidentalis*. Unions of the resulting *F*_ST_ outlier lists were taken to generate final lists of candidate loci.

### 2.7 Genotype–environment associations

To determine whether variation in environmental conditions is related to genomic variation within the spruce budworm species complex, we used latent factor mixed modeling (LFMM) to assess correlations between allele frequencies and environmental conditions across sampling locations. It is possible that both *C*. *fumiferana* and *C. occidentalis* share ancestral adaptations to environmental conditions and may therefore exhibit the same genotype–environment associations. Alternatively, these species may be adapted to variation in environmental conditions in different ways and exhibit different genotype–environment associations. We therefore quantified genotype‐environment associations for all *Choristoneura* individuals together (*C*.*fumiferana* + *C*. *o. biennis* + *C. o. occidentalis*), *C. occidentalis* independently (*C. o. biennis* + *C. o. occidentalis*), and *C. fumiferana* independently. This was accomplished using LFMM 2 (Caye et al., 2019) implemented via the R package LEA (Frichot & François, 2015). LFMM 2 implements factor regression on a genotypic matrix using local environmental conditions as explanatory variables, while controlling for background population structure using latent factors (using an exact least‐squares approach). This reduces the likelihood of false positives arising from autocorrelation of demography, space, and environmental conditions (Caye et al., 2019; Frichot et al., 2013; Lotterhos & Whitlock, 2014).

Environmental variables were compiled from the Worldclim 2 database (Fick & Hijmans, 2017), including mean annual temperature, mean temperature of warmest month, mean temperature of coldest month, mean temperature of warmest quarter, mean temperature of coldest quarter, mean annual precipitation, mean precipitation of warmest quarter, and mean precipitation of coldest quarter. For all *Choristoneura* individuals together (*C*. *fumiferana* + *C*. *o. biennis* + *C. o. occidentalis*), *C. occidentalis* independently (*C. o. biennis* + *C. o. occidentalis*), and *C. fumiferana* independently, we completed LFMM 2 analysis using latent factors equal in number to the optimal value of *k* inferred from *structure* analyses. The FDR criterion was again used to control for false positives associated with multiple tests with a *q*‐value threshold of 0.05 (Benjamini & Hochberg, 1995). For loci with multiple significant environmental associations (likely due to spatial correlation of environmental variables), we determined the strongest association based on median |*z*|‐scores (MacDonald et al., 2020; Martins et al., 2018).

### 2.8 Genomic locations and identities of SNPs

We performed BLASTN searches using flanking sequences around candidate/outlier loci derived by BayeScan and LFMM 2 to determine putative SNP identity. We used the Larroque et al. (2019) draft genome to retrieve 5 kb on either side of each SNP, or all bases between the SNP position and the end of the scaffold if it was within 5 kb. We then searched NCBI’s nonredundant sequence databases using BLASTN version 2.11.0 to find nucleotide matches for these regions (Madden, 2002). We inferred the function of the top sequence matches using the UniProt database (The Uniprot Consortium, 2019). Additionally, we determined the location of these SNPs on the *C. fumiferana* linkage map (Picq et al., 2018) to evaluate whether major genetic differences between and within these taxa are restricted to linkage groups.

## 3 RESULTS

### 3.1 Specimen collections and DNA data processing

A total of 1011 and 7276 adult male *Choristoneura* were collected during the 2017 and 2018 collection seasons, respectively (Figure 1; Tables S2,S3,S6). Flight peaks were observed by early or mid‐July across all nine locations in 2017 (mainly collection interval 4, the ten days prior to July 13–17 in 2017; Table S3). In 2018, flight peaks were observed in early August at several locations, and the flight period was longer in the 2017 season (Figure 1). No *Choristoneura* were captured in a control trap at any location.

We filtered raw Illumina‐generated reads from 267 *Choristoneura* specimens in a preliminary run of Stacks 2. We removed five individuals with >30% missing data and reran Stacks 2 with 262 individuals. Two more individuals were removed after re‐running Stacks 2; one with >30% missing data in the re‐run, and the other with divergent placement in PCA, which we suspect represented another third species of *Choristoneura*. Within this final set of 260 individuals, *gstacks* retained 340,937 loci across 114 million reads (mean of ~434,100 reads aligned per specimen). These loci were further filtered in *populations* to obtain 13,972 SNPs; final filtering in VCFtools resulted in a dataset of 2831 SNPs with an average read depth of 15.7x and 2.79% missing data.

### 3.2 Identity of individuals

**FIGURE 2:**
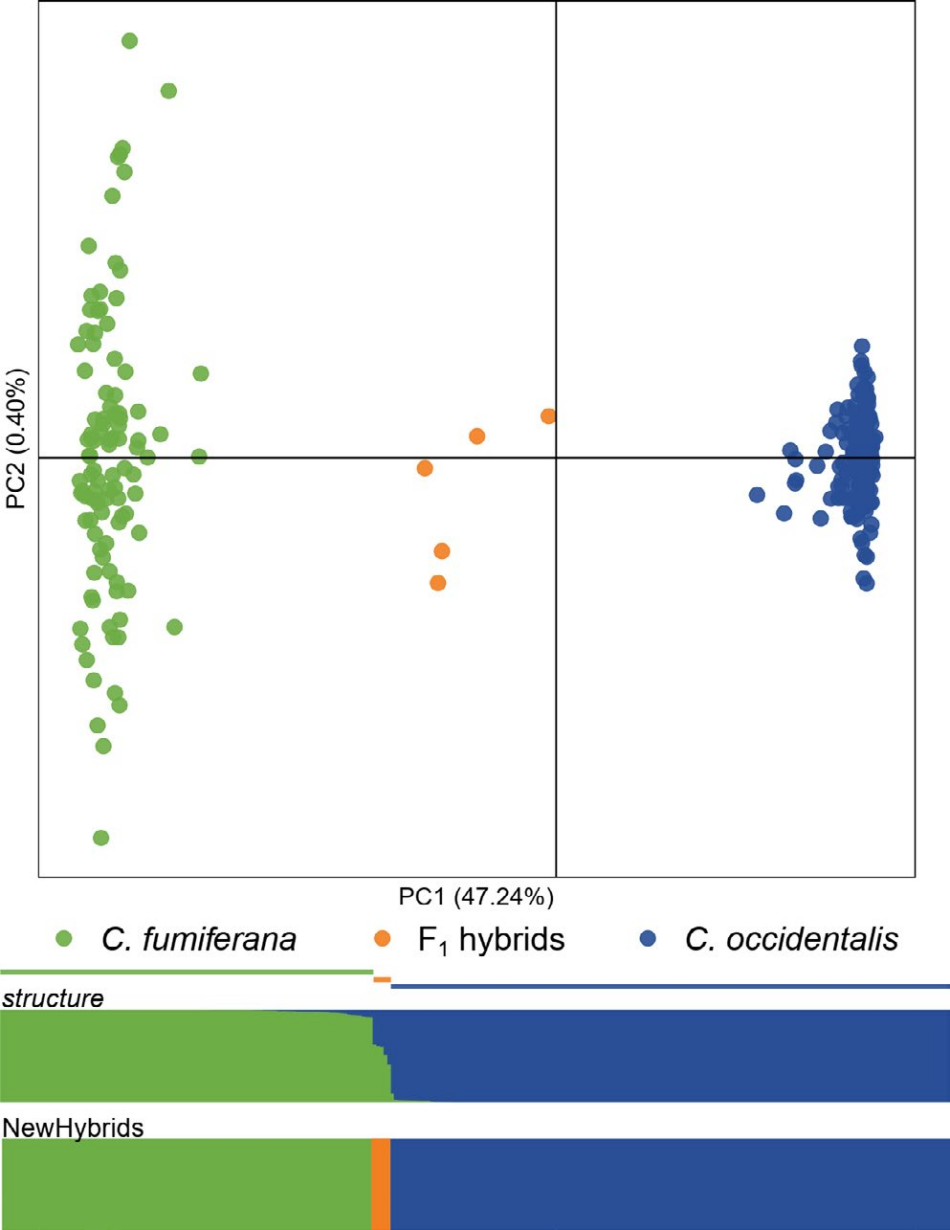
Principal component analysis, *structure* barplot, and NewHybrids barplot of SNPs for 260 genotyped *Choristoneura*. Individuals with <90% assignment to either cluster in the *structure* analysis are labeled “F_1_ hybrids” based on NewHybrids assignment

Two major genetic clusters and a small number of intermediates were evident in principal component analysis (Figure 2). Genetic clusters were identified with *structure* admixture coefficients; 102 individuals were identified as *C. fumiferana* and were genotyped in similar numbers each year (*n* = 54 in 2017, *n* = 48 in 2018) and 153 individuals were identified as *C. occidentalis* (sensu Brunet et al., 2017), 84% of which were collected in 2018 (*n* = 25 in 2017, *n* = 128 in 2018). We found greatest support for two genetic clusters among the 260 individuals using both the Δ*k* method (Evanno et al., 2005) and the rate of change in the likelihood of *k* from 1–10 (Pritchard et al., 2000, see Figures S1 and S2). Five individuals in an intermediate third genetic group were identified as F_1_ hybrids based on NewHybrids assignment using both the first 400 SNPs (Figure 2) and 400 randomly chosen SNPs (not shown). We estimated a hybridization rate of 2.9% for *C. fumiferana* and *C. occidentalis* in their overlapping range (Table 1) based on the proportion of F_1_ hybrid individuals relative to those in the two parental genetic clusters (locations d–j, Figure 1). Records of all genotyped individuals are listed in Table S7.

**TABLE 1:** Estimates of hybridization rates between *C. fumiferana* and subspecies of *C. occidentalis* in Alberta

Location	Method to determine hybridization rate	
	Microsatellite / mtDNA mismatch	ddRADseq F_1_ ^e^
Cypress Hills, Alberta^a^	**7.3%** (21 of 286)	–
Crowsnest Pass, Alberta^b^	**7.0%** (3 of 41)	–
Canadian Rocky Mountains and western Alberta^c^	–	**0.6%** (3 of 529)
range overlap of *C. fum* and *C. o. bie* + *C. o. occ* ^c^	–	**1.1%** (3 of 281)
range overlap of *C. fum* and *C. o. bie* + *C. o. occ* ^d^	–	**2.9%** (5 of 174)

^a^ Lumley & Sperling 2011a;

^b^ Brunet et al. 2013;

^c^ Brunet et al. 2017;

^d^ Present study.

^e^ Approximately 50% membership to both *C. fumiferana* and *C. occidentalis* genetic clusters in *structure*, or F_1_ assignment in NewHybrids.

Hierarchical *structure* analyses suggested the existence of two indistinct genetic clusters within *C. occidentalis* (Figures S3–S5). Individuals assigned to the largest cluster (*n* = 147) were identified as *C. o*. *biennis* in accordance with Brunet et al. (2017). The six remaining individuals were identified as *C. o*. *occidentalis*, which were primarily collected at Clearwater in British Columbia (location a). There was no apparent substructure within *C. fumiferana* based on either PCA or hierarchical *structure* analysis (Figure S6). This was supported by incongruence between the LnP(*k*) and Δ*k* methods, in addition to low support for any one Δ*k* value (Figures S7 and S8; *structure* plot not shown).

### 3.3 Temporal separation between and within species

**FIGURE 3:**
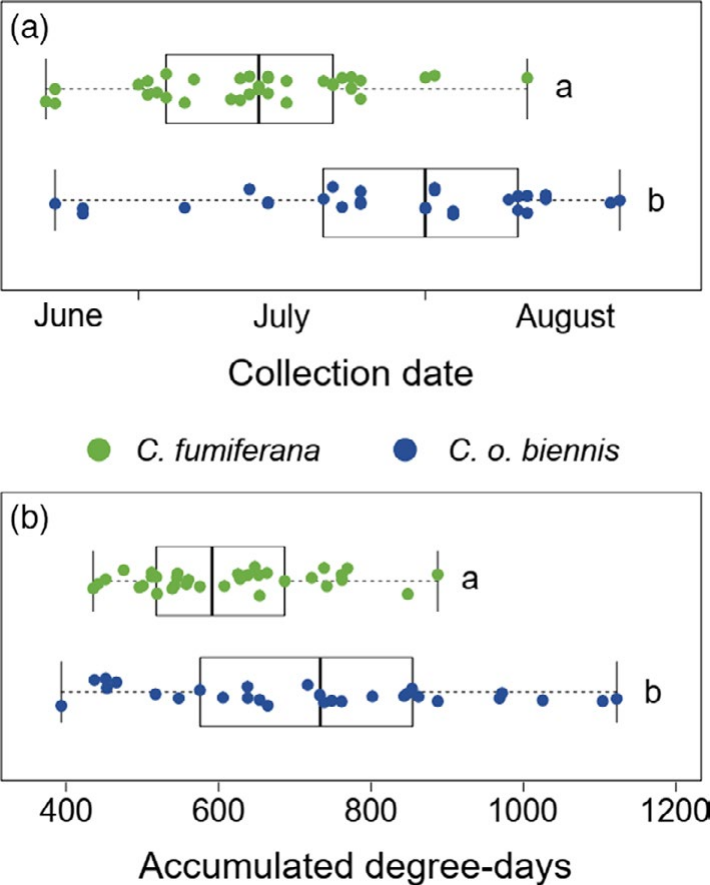
Boxplots for median collection dates (a) and accumulated degree‐days (b) for adult *Choristoneura fumiferana* and *C. o. biennis* in region of overlap (locations d–j) during 2017 and 2018. Both had significant differences in maximum flight abundance (Wilcoxon–Mann–Whitney tests: collection date: U = 5151, *p* < 0.001, *r* = 0.67; accumulated degree‐days: U = 1683, *p* < 0.001, *r* = 0.42). Error bars depict standard error

**FIGURE 4:**
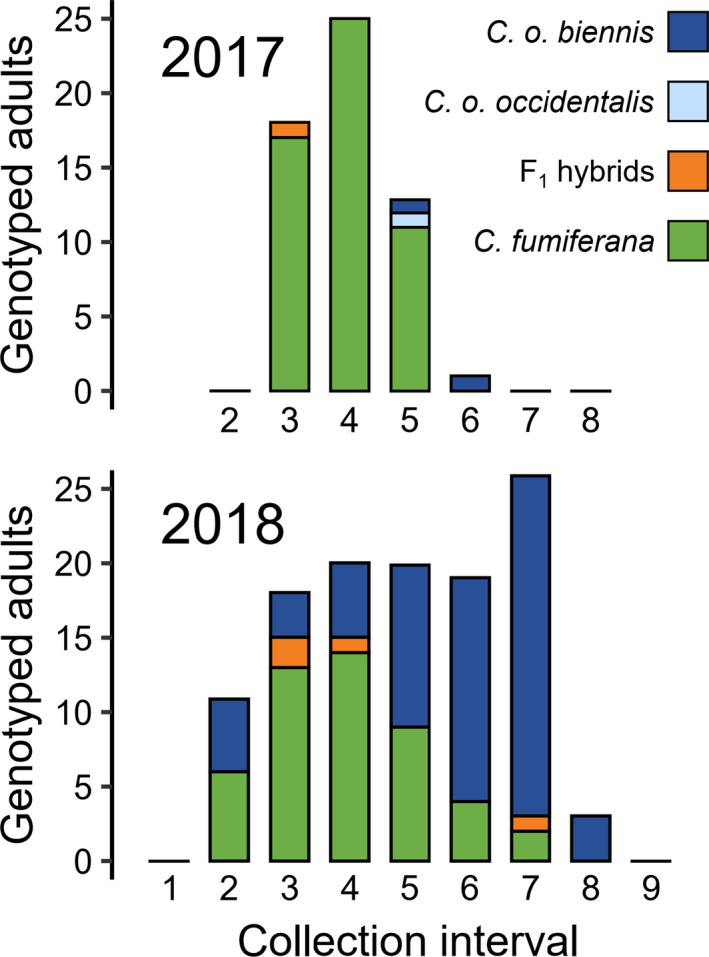
Pooled numbers of genotyped adult *Choristoneura fumiferana* and *C. occidentalis* from zone of sympatry (locations d–j). Histograms show numbers of adult *Choristoneura* genotyped from each collection interval, with traps being emptied in the date ranges listed for Figure 1 (Tables S2 and S3). Traps were not deployed for 2017 collection intervals 1 and 9

**FIGURE 5:**
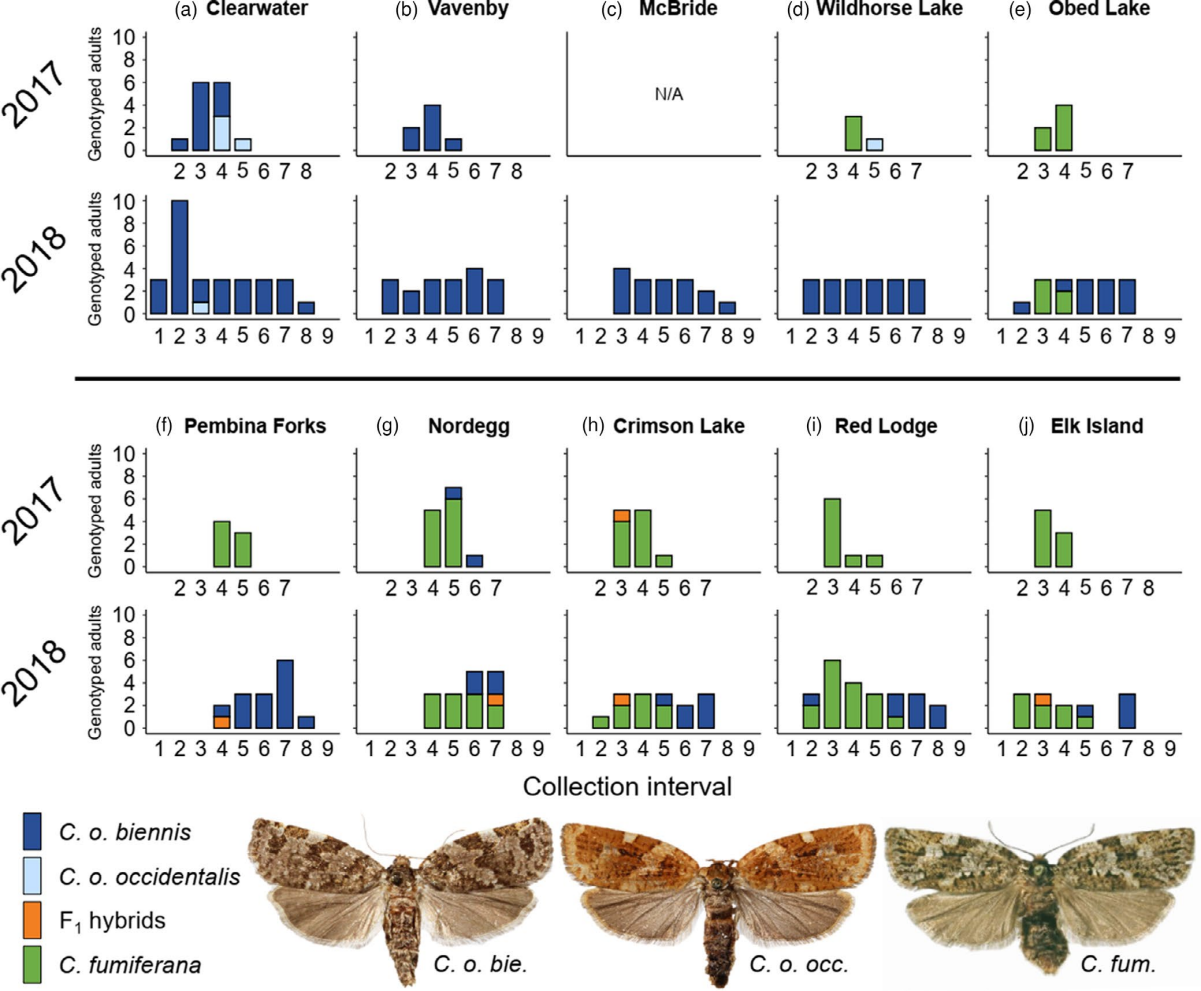
Number of genotyped adult *Choristoneura* from each collection location. Moths were pheromone‐trapped at each location during ~10 days before collection. Histograms show numbers of adult *Choristoneura* trapped during each collection interval, with traps being emptied in the date ranges listed for Figure 1 (Tables S2 and S3). Collection intervals without number digits from 2017 indicate traps were not deployed, while numbered collection intervals without histogram bars indicate no moths were trapped

The median flight dates of genotyped *C. fumiferana* and *C. o*. *biennis* differed by approximately three weeks within their zone of sympatry (Figures 3 and 4; U = 5151, *p* < 0.001, *r* = 0.67). Degree‐day accumulation before peak flight also differed significantly between species (U = 1683, *p* < 0.001, *r* = 0.42). However, temporal isolation was incomplete at all locations where both species occurred (Figure 5; locations d–j). Our first co‐occurrence index, calculated as the number of collection periods in which both *C. fumiferana* and *C. occidentalis* were present divided by the total collection periods that either species was present, estimated a location‐by‐location co‐occurrence rate of 12.4% and a pooled‐location co‐occurrence rate of 63.6%. Similarly, our second co‐occurrence index, accounting for the relative abundance *C. fumiferana* and *C. occidentalis*, estimated a location‐by‐location co‐occurrence rate of 16.0% and a pooled‐location co‐occurrence rate of 72.8%.

In Alberta, genotyping revealed a substantial increase in the abundance of *C. o. biennis* at Wildhorse Lake and Pembina Forks from 2017 to 2018 (Figure 5, locations d and f). Although only Nordegg (location g) had *C. o. biennis* in 2017, all Alberta locations had some *C. o. biennis* in 2018, and these individuals usually flew later in the season. The last four individuals genotyped at Elk Island (location j) in the 2018 flight season were *C. o. biennis*, the location where we expected to find only *C. fumiferana*. In British Columbia, we collected both subspecies of *C. occidentalis* at Clearwater (location a), although *C. o. biennis* was the most common (Figure 5). Specimens collected at Vavenby and McBride (locations b and c) did not include *C. o. occidentalis*. One *C. o. occidentalis* was identified in the overlap region of Alberta (Wildhorse Lake, location d, Figure 5). Too few *C. o. occidentalis* were sampled to confidently assess its flight period separately from *C. o. biennis*, although the individuals genotyped from Clearwater in 2017 were found in the later part of the flight season of that year.

### 3.4 Genomic regions with high divergence

BayeScan identified 96 loci (3.4% of 2831 SNPs) with *F*_ST_ values that were significantly higher than background divergence between *C. fumiferana* and *C. occidentalis* (Table S8). Mean *F*_ST_ of these SNPs was 0.70 (standard deviation (SD) = 0.046), while overall *F*_ST_ between the species was 0.34 (SD = 0.33). Within *C. occidentalis*, BayeScan identified 4 *F*_ST_ outlier loci (0.28% of 1416 SNPs) between *C. o. biennis* and *C. o. occidentalis* (Table S9). Mean *F*_ST_ of these loci was 0.17 (SD = 0.035), and overall *F*_ST_ between the subspecies was 0.01 (SD = 0.08).

### 3.5 Genotype–environment associations

LFMM 2 identified two loci that were significantly associated with mean precipitation of warmest quarter (all *Choristoneura*, Figure S9, Table S8) and mean annual precipitation (*C. fumiferana* independently, Figure S10, Table S10). Neither of these loci were identified as significant *F*_ST_ outliers by BayeScan (Tables S8 and S10). No significant genotype–environment associations were detected when addressing *C. occidentalis* independently (*C. o. biennis* + *C*. *o. occidentalis*) (Figure S11).

### 3.6 SNP identity and genomic location

Together, BayeScan and LFMM 2 identified 97 loci that may represent genomic regions under selection in the *C. fumiferana* and *C. occidentalis* dataset (Table S8). Top BLASTN search results suggest that 17 of these loci are associated with genes encoding antifreeze proteins and 1 with a pheromone/odorant binding gene. Many other matches were *C. o. occidentalis* microsatellite sequences or uncharacterized beyond mRNAs or draft genome scaffolds of other Lepidoptera. Thirty‐seven of 97 locus flanking sequences had matches on the *C. fumiferana* linkage map (Picq et al., 2018); 25 loci on linkage group Z (the male sex chromosome) and <4 loci on linkage groups 3, 4, 5, 6, 8, 12, 13, and 25 (Table S8). In the *C. o. biennis* and *C. o. occidentalis* dataset, 4 *F*_ST_ outliers were identified by BayeScan (Table S9). Of these, 2 were associated with genes encoding antifreeze proteins. One locus flanking sequence had a match on the *C. fumiferana* linkage map on linkage group 16 (Picq et al., 2018). In the *C. fumiferana* dataset, 1 locus was identified by LFMM 2 as having a significant environmental association (Table S10). Its flanking sequence had no match on the *C. fumiferana* linkage map (Picq et al., 2018).

## 4 DISCUSSION

### 4.1 Temporal separation and isolation between species

Our study demonstrates substantial, but incomplete, phenological divergence between sympatric *C. fumiferana* and *C. occidentalis*. This trend is apparent in either pooled‐location or location‐by‐location evaluations of temporal relationships among closely related taxa. When pooling individuals from all locations, temporal overlap between species occurred in most (63.6%) collection intervals over two years (Figure 4). In contrast, temporal overlap was observed in far fewer intervals on a location‐by‐location basis (12.4%; Figure 5). Similar estimates of temporal overlap were found using our second co‐occurrence index that accounted for the abundance of each species. Together, these results suggest that combining species occurrence records across a broad spatial extent overestimates the actual temporal overlap of sympatric populations. The extent of temporal contact between these species also varied substantially between years, highlighting the need to consider variation in voltinism in these investigations. In 2017, *C. fumiferana* and *C. o. biennis* were both collected in the same collection interval at only one of seven possible collection locations. In 2018, these taxa were both collected in the same collection interval at five of the seven collection locations, including at Elk Island where we initially expected to find only *C. fumiferana* (Figure 5; Dupuis et al., 2017; Lumley & Sperling, 2011b). Our results clearly show that the greatest contact between these taxa occurs every second year, when most *C. o. biennis* emerge as adults. However, in both 2017 and 2018, peak flights of *C. fumiferana* were earlier than those of *C. o. biennis* at each sympatric collection location (Figure 5). This pattern was maintained when individuals from all sympatric collection locations were pooled for flight peak comparisons (Figure 4). Because semivoltine *C. o. biennis* did not fly before univoltine *C. fumiferana* in years of greatest contact, we can infer that their biennialism does not contribute to a phenological “head start” relative to sympatric *C. fumiferana*.

We observed two instances wherein *C. o. biennis* was detected in its region of sympatry with *C. fumiferana* at the beginning of the collection season in late June, much earlier than the taxon's regional flight peak (Figure 5, Obed Lake and Red Lodge). It is possible that these two individuals eclosed in British Columbia in late June and were blown into Alberta via prevailing winds, as their calendar‐day phenology matches that of *C. o. biennis* from Clearwater and Vavenby (see Lumley et al., 2020). Such dispersal may also explain why the frequency of genotyped *C. o. biennis* was unusually high at Wildhorse Lake and Pembina Forks locations, which are closer to the main range of this species (Figure 1). *Choristoneura* are known to disperse great distances; Dobesberger et al. (1983) found that *C*. *fumiferana* can travel more than 450 km under certain conditions and that these long‐distance dispersal events are common during outbreaks of *C. fumiferana* in eastern North America (Greenbank et al., 1980; Sturtevant et al., 2013). Results from *structure* reinforce this prevailing‐wind hypothesis and suggest that gene flow between these species is asymmetric; more *C. fumiferana* individuals exhibit some membership to the *C. occidentalis* genetic cluster than *C. occidentalis* individuals to the *C. fumiferana* cluster (Figure 2; and see Brunet et al., 2017).

Our repeated sampling across consecutive years helps clarify temporal and spatial relationships between *C. fumiferana* and *C. occidentalis*. Previously, range‐wide sympatry of these species has been explored without explicit consideration of their temporal interactions (Brunet et al., 2017; Dupuis et al., 2017; Lumley & Sperling, 2011b), despite the known biennialism of *C. o. biennis*. We found that *C. o. biennis* extends at least to Elk Island, Alberta (Figure 5), but this was only apparent because sampling was completed in consecutive years. It is possible that these adults dispersed from areas adjacent or even distant from their collection locations after developing and eclosing under different ecological and environmental conditions (Silva‐Brandão et al., 2015). Indeed, adult *C. fumiferana* collected in Unitraps are not always from the same population as larvae feeding at the same location (James et al., 2015). Likewise, the *C. o. occidentalis* collected in both years from Clearwater, British Columbia, were outside of their known range but within plausible dispersal distance from suitable habitat. We find that adult occurrences of both *C. o. biennis* and *C. o. occidentalis* span a larger geographic area than previously documented. However, we cannot be sure whether these are vagrant individuals or representatives of local, reproducing populations. We therefore recommend that future studies should compare larval and adult genotypes in the zone of sympatry between *C. fumiferana* and *C. occidentalis* to better understand the reproductive range of each species, and their interaction with host conifers.

### 4.2 Extent of hybridization between species

Several studies have demonstrated that *Choristoneura* taxa can hybridize freely in laboratory settings (Harvey, 1997; Nealis, 2005; Sanders et al., 1977). However, our SNP‐based identifications of wild specimens allowed us to confirm that hybrid adults are rare in their zone of sympatry, despite some degree of reproductive overlap. Using NewHybrids, we estimated a hybridization rate of 2.9% for *C. fumiferana* × *C. o. biennis* (Table 1). Previous studies have found hybridization rates of 0.6 and 7% between *C. fumiferana* and various subspecies of *C. occidentalis*, based on proportions of hybrids detected using genomic data (Table 1; Brunet et al., 2013, 2017; Lumley & Sperling, 2011a). However, these studies did not sample at temporal or spatial scales sufficient to confidently assess hybridization across entire flight periods, as each was focused on resolving taxonomic relationships within *Choristoneura*, rather than the ecology and evolution of component species.

Selection against hybrid individuals exhibiting intermediate developmental rates could explain why few hybrid adults were detected in this study and why they are commonly collected near the peak flight of either parental taxon, rather than between flight period peaks. Hybrid larvae with intermediate developmental rates may miss their feeding “window of opportunity” (sensu Lawrence et al., 1997; Fuentealba et al., 2018). Past research has shown that larvae of conifer‐feeding *Choristoneura* are in tight temporal synchrony with the annual bud flush of their host plant (Lawrence et al., 1997; Marshall & Roe, 2021; Nealis, 2012; Volney & Cerezke, 1992). If larvae develop too slowly, their likelihood of achieving adulthood drops precipitously (Lawrence et al., 1997). Larval *C. fumiferana* × *C*. *o. biennis* hybrids with intermediate developmental rates may emerge from their hibernacula too late or too early to successfully feed on their host plants at a given location. Even though white spruce, Engelmann spruce, and subalpine fir can have similar phenologies at locations where they occur together, bud flush often differs by several weeks at different elevations (Coates et al., 1994), putting intermediate larvae at a disadvantage at any one location. F_1_ hybrids may also be infertile. All hybrid individuals were assigned to the F_1_ hybrid class with >99% certainty, with no F_2_ or backcrossed offspring detected. This may be evidence that hybrid breakdown between these species is underway (Sperling, 1994). Hybridization of these *Choristoneura* species in laboratory settings will be required to resolve whether F_1_ offspring indeed exhibit intermediate developmental rates and if they are infertile.

### 4.3 Potential environmental adaptations

*F*_ST_‐based outlier (Bayescan) and genotype–environment association (LFMM 2) analyses identified several genomic regions that may be under selection within the spruce budworm species complex. Many of the regions flanking loci identified by BayeScan for both the *C. fumiferana* + *C*. *occidentalis* and *C. o. biennis* + *C. o. occidentalis* datasets had putative associations with antifreeze proteins (Tables S8 and S9). These proteins encapsulate ice crystals within *Choristoneura* larvae during cold periods, aiding in their overwintering success by lowering their hemolymph freezing point (Marshall & Roe, 2021; Tyshenko et al., 1997). Such candidate loci under selection that confer overwintering success may have helped *C. o. biennis* adapt to cooler environments, as this subspecies is typically found at higher elevations and latitudes than the other taxa (Dupuis et al., 2017; Shepherd et al., 1995).

Despite the reduced‐representation methods applied here, wherein only a small fraction of individuals’ genomes are sequenced, we still detected a few significant associations between genomic variation and environmental conditions within the spruce budworm species complex. The LFMM 2 candidate SNP in the *C. fumiferana* + *C*. *occidentalis* dataset was strongly associated with both mean temperature and precipitation of the warmest quarter of the year, during which *Choristoneura* diapause is initiated (Han & Bauce, 1998). Together, Bayescan and LFMM 2 results suggest that ecologically mediated selection and temporal isolation may be synergistically contributing to evolutionary divergences within the spruce budworm species complex and that either mechanism in isolation cannot account for reproductive isolation. We suggest that future work, using whole‐genome sequencing techniques, should be completed to identify additional genomic regions that may be implicated in divergent selection among taxa and local adaptation to environmental conditions.

### 4.4 Genomic architecture of Choristoneura

Our finding that 26 of 37 *F*_ST_ outliers between *C. fumiferana* + *C*. *occidentalis* had linkage group matches on the Z sex chromosome (Table S8) suggests that adaptive divergence within *Choristoneura* is disproportionately sex‐linked (Sperling, 1994). Sex‐linked genes are often greater contributors to species‐level divergence than autosomal genes (Baiz et al., 2020; Janz, 2003; Presgraves, 2018) and are known to evolve more quickly due to their hemizygous state in one sex (“faster X evolution”, Bachtrog et al., 2009; Charlesworth et al., 1987). Further, several of the Z‐linked candidate loci were associated with antifreeze genes that may contribute to overwintering success. Sex chromosomes of other Lepidoptera have been found to house many adaptive loci associated with diapause (Fu et al., 2015; Pruisscher et al., 2018, 2020), demonstrating their importance for rapid evolution of the trait. Indeed, diapause frequency is the only consistent phenotypic difference that separates *C. o. biennis* from other conifer‐feeding *Choristoneura*. However, it is also possible that higher rates of genetic drift associated with lower effective population sizes and the absence of recombination could account for high *F*_ST_ values observed on the Z sex chromosome (Charlesworth et al., 1987; Ravinet et al., 2017). Further investigation of divergence of coding regions on the Z sex chromosome, such as those associated with diapause, are required to advance our understanding adaptive divergence in the *C. fumiferana* species complex.

### 4.5 Applications to forest management

Our study effectively quantified temporal and spatial relationships between *C. fumiferana* and *C. occidentalis* across their zone of sympatry, demonstrating that phenological divergences are sufficiently large to contribute to reproductive isolation between the species. However, some degree of phenological overlap was still observed between the species, providing opportunity for hybridization. Both *C. fumiferana* and *C. occidentalis* are destructive defoliators of conifers throughout North America, and several phenological models have been developed to detect and manage their outbreaks (e.g., Régnière, 1990; Régnière & Nealis, 2018; Régnière et al., 2012). *Choristoneura fumiferana* models have generally been parametrized using occurrence and phenological records from Ontario and Quebec, Canada, and extrapolated to the western edge of the species’ range, but with reduced predictive power (Candau et al., 2019; Régnière et al., 2012). Similarly, *C. occidentalis* models have been parameterized far from the zone of *C. fumiferana*–*C. occidentalis* sympatry (Nealis & Régnière, 2014; Régnière & Nealis, 2018). Outbreak models would benefit from the inclusion of occurrence and phenological data from the western and eastern edges of ranges of *C. fumiferana* and *C. occidentalis*, respectively, where the two species are sympatric. Our findings show that, due to overlap of flight periods within this zone of sympatry, species identity cannot be assigned based on collection time. Individuals will need to be identified using genomic data to effectively parameterize outbreak models. Additionally, given that *C. fumiferana* and *C. occidentalis* are likely to increase their zone of sympatry in the northern boreal forest by mid‐century (Régnière & Nealis, 2019; Régnière et al., 2012), understanding their potential for introgression and mechanisms of reproductive isolation gives insight into how they will interact at higher latitudes. Hybridization is likely to occur across an increasingly larger zone of sympatry as climatic conditions change, but we predict that these species will maintain their genomic integrity as they have in their present‐day zone of sympatry.

## CONFLICT OF INTEREST

We declare no conflict of interest.

## Supporting information

Fig S1‐S11

Table S1‐S10

## Data Availability

DNA sequences are available in fastq format in the National Center for Biotechnology Information Sequence Read Archive (NCBI SRA) under the accession number PRJNA798113
