# Supplementary material for: Moths passing in the night: Phenological and genomic divergences within a forest pest complex
Source: Evol Appl. 2022 Jan 11;15(1):166–80. doi: 10.1111/eva.13338 (PMC8792478; doi:10.1111/eva.13338)
Supplement: Supplementary file 1 — Fig S1‐S11 [file EVA-15-166-s001.docx]

Supplemental info I:

Moths passing in the night: phenological and genomic divergences within a forest pest complex


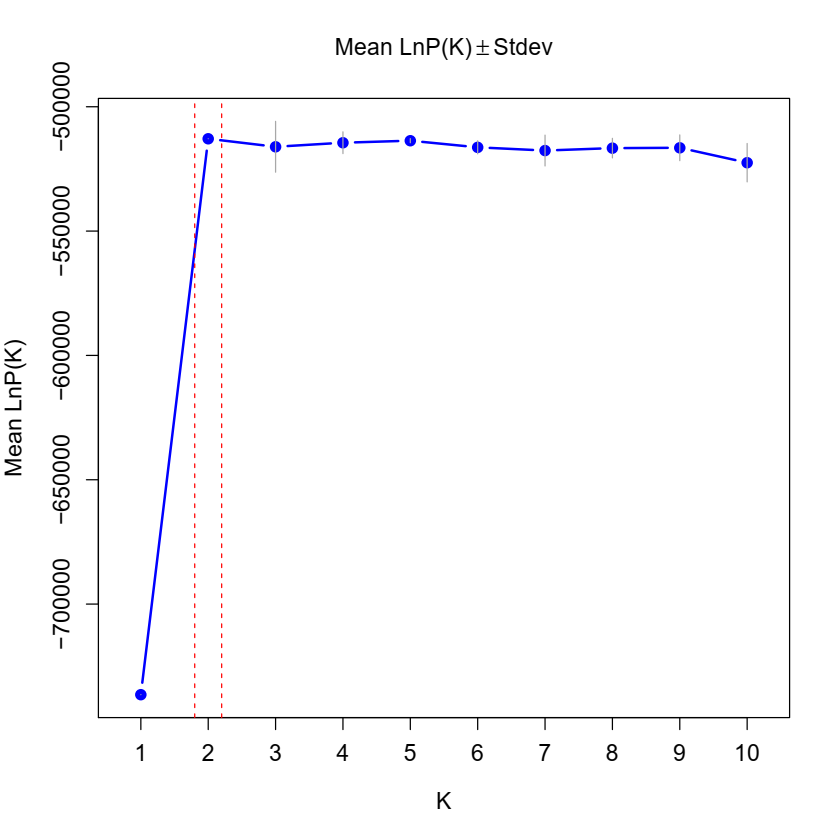


**Supplemental Figure 1.** Support for LnP(*k*) 1–10 for the *Choristoneura fumiferana* + *Choristoneura occidentalis* dataset.


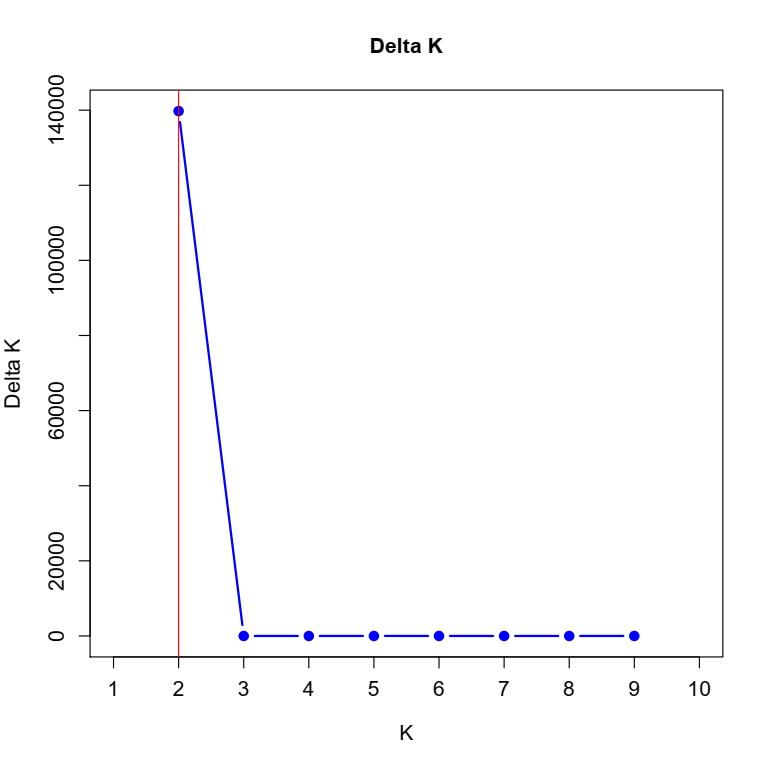


**Supplemental Figure 2.** Support for Δ*k* 2–9 for the *Choristoneura fumiferana* + *Choristoneura occidentalis* dataset.


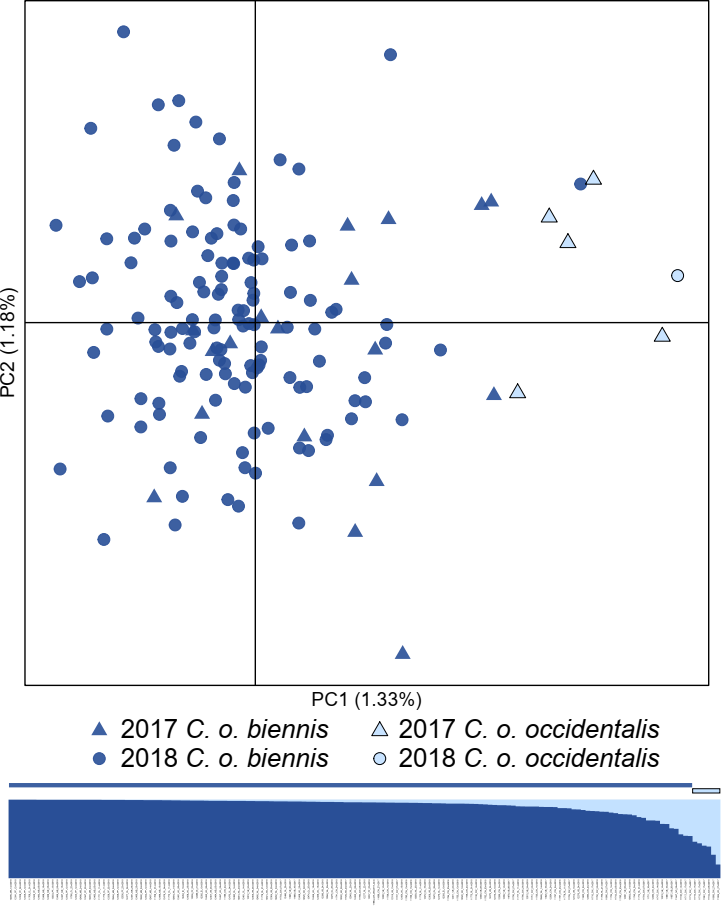


**Supplemental Figure 3.** Principal component analysis and *structure* barplot of SNPs for 153 *C. occidentalis* individuals. Individuals with <50% assignment to the major cluster (*C. o. biennis*) in the structure plot are to the right in the PCA, light blue, and labelled “*C. o. occidentalis*”. Year of collection is indicated by symbol shape (triangle = 2017, circle = 2018).


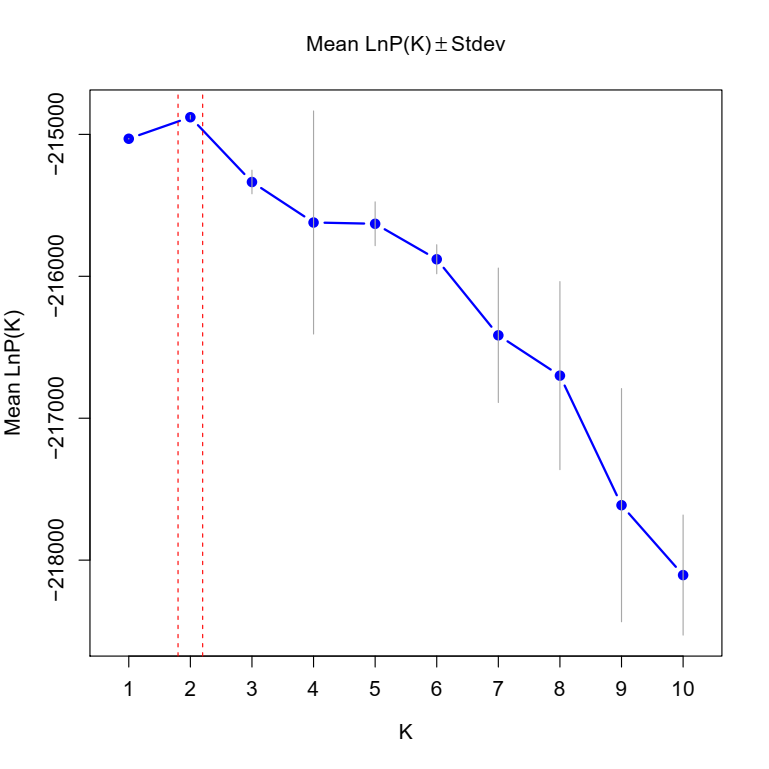


**Supplemental Figure 4.** Support for LnP(*k*) 1–10 for the *Choristoneura occidentalis biennis* + *Choristoneura occidentalis occidentalis* dataset.


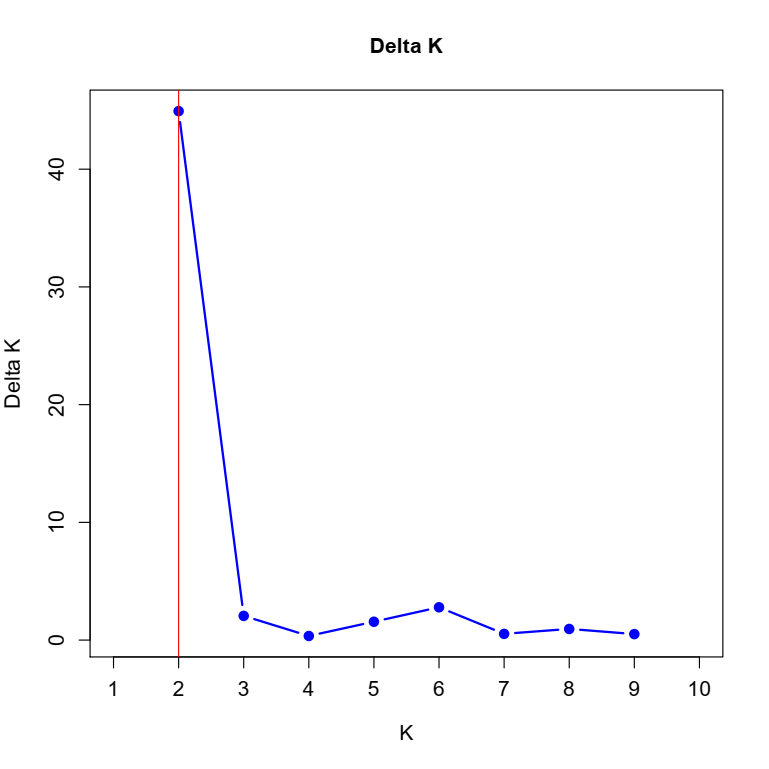


**Supplemental Figure 5.** Support for Δ*k* 2–9 for the *Choristoneura occidentalis biennis* + *Choristoneura occidentalis occidentalis* dataset.


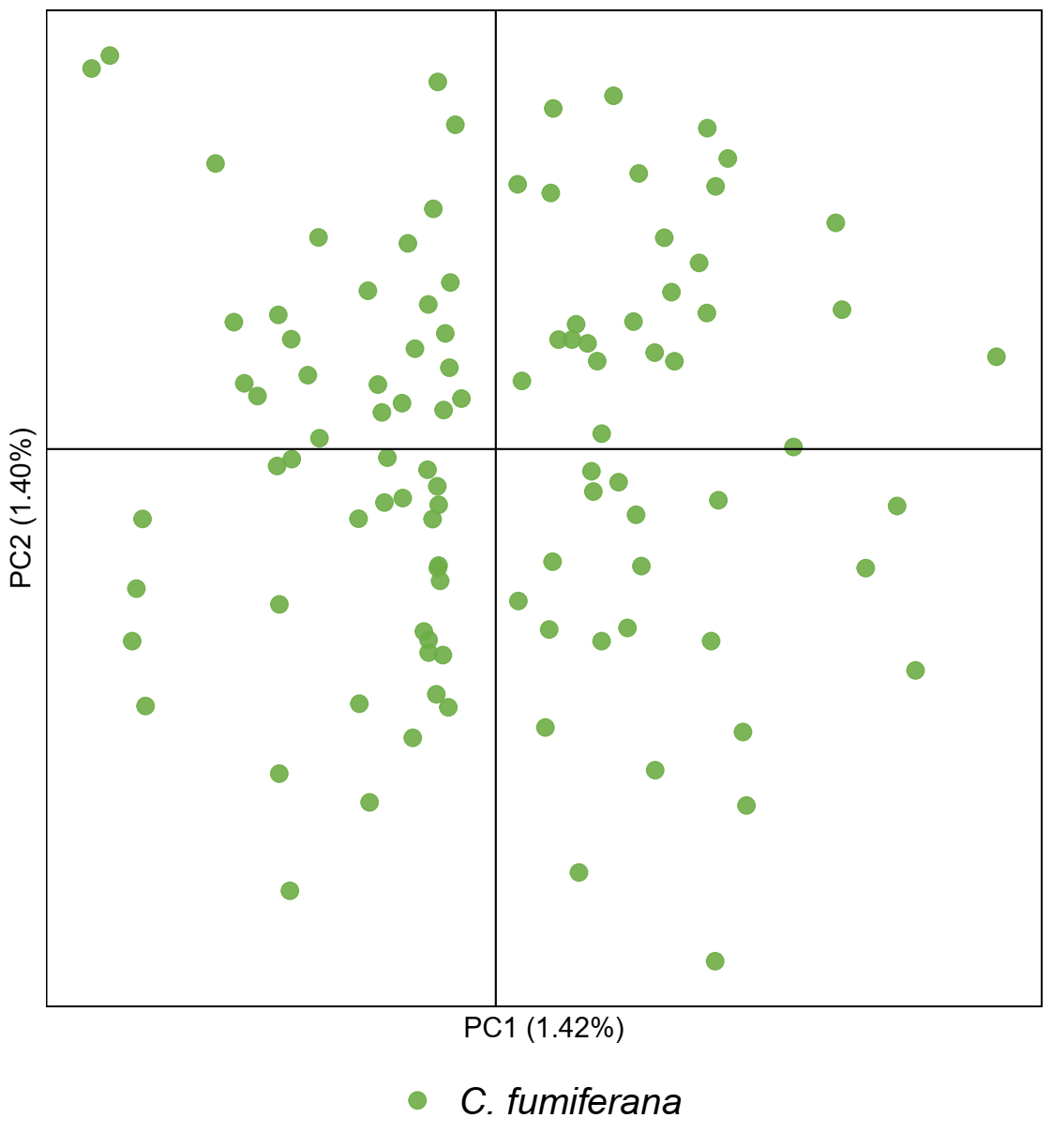


**Supplemental Figure 6.** Principal component analysis of SNPs in 102 *Choristoneura fumiferana* individuals*.*


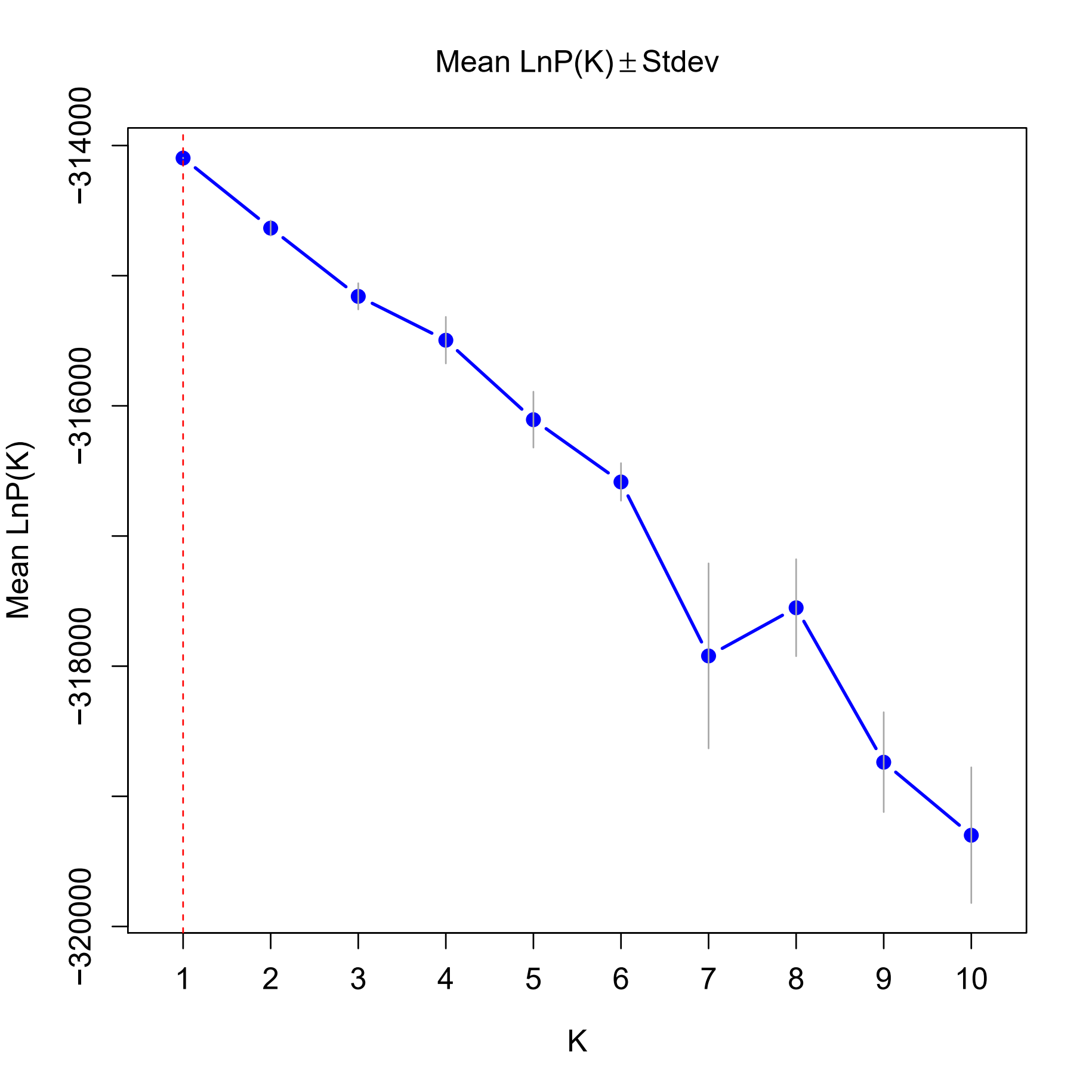


**Supplemental Figure 7.** Support for LnP(*k*) 1–10 for the *Choristoneura fumiferana* dataset.


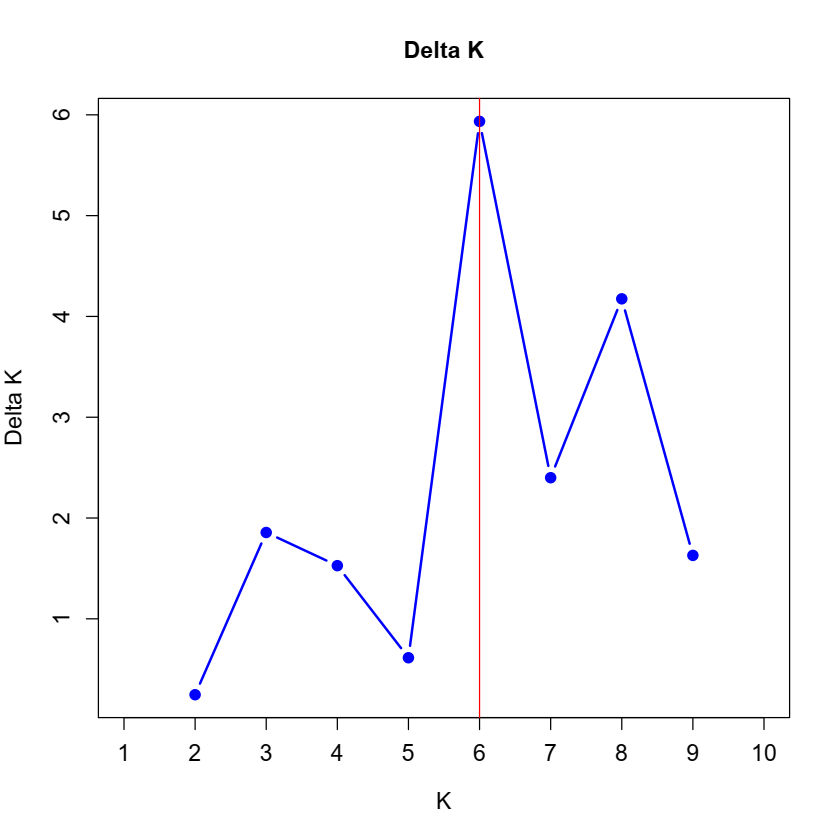


**Supplemental Figure 8.** Support for Δ*k* 2–9 for the *Choristoneura fumiferana* dataset.


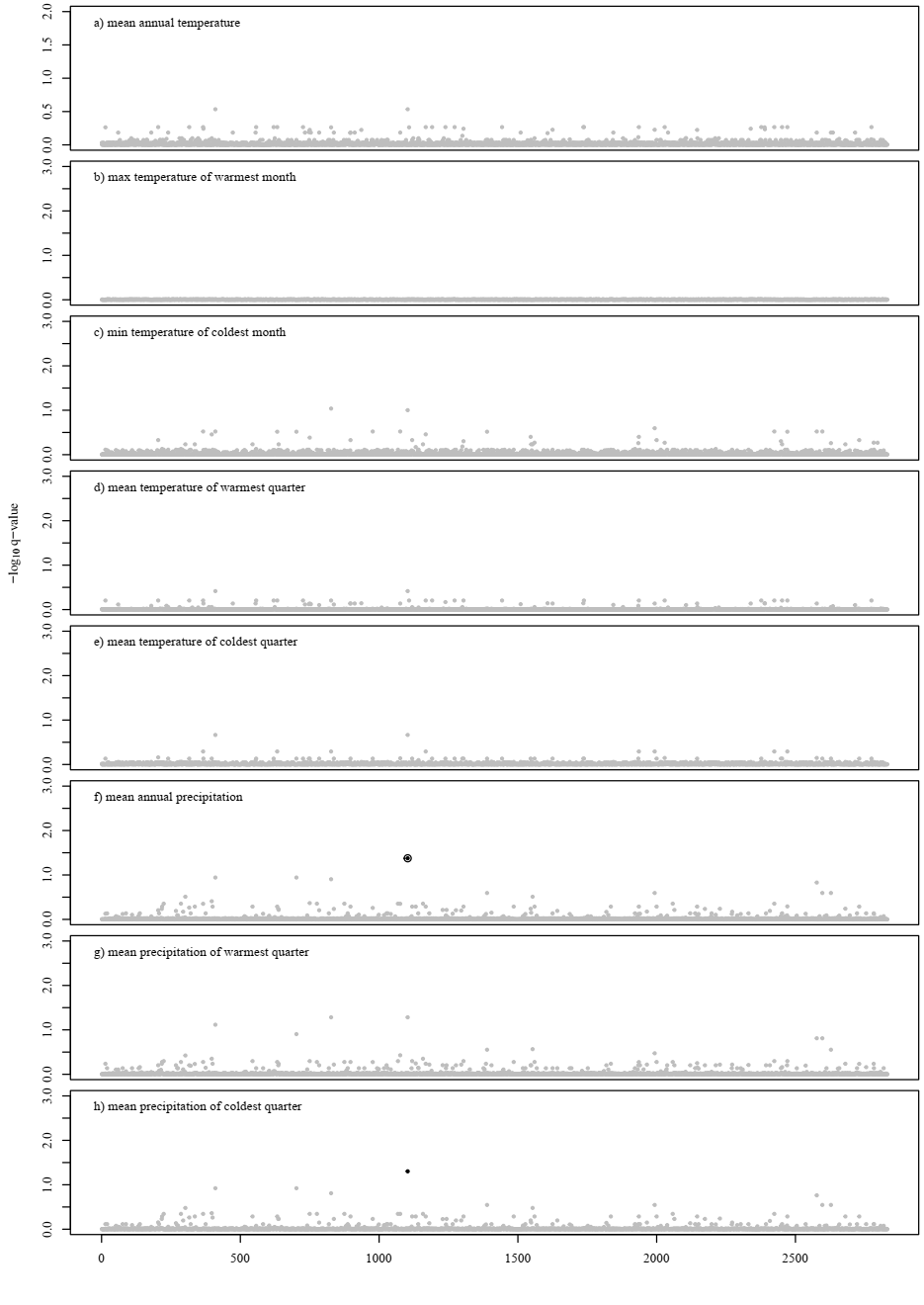


**Supplemental Figure 9.** Candidate SNPs associated with Worldclim 2 database variables derived from latent factor mixed models of *C. fumiferana* and *C. occidentalis* collected in Alberta and British Columbia. Black dots indicate loci with significant associations to the relevant environmental variable based on a *q*-value threshold of 0.05 (−log_10_ *q*-value ~1.3). Single loci often had multiple significant environmental associations due to spatial correlation of environmental variables. Open circles around black dots represent the strongest association (based on median |z|-scores) for each locus with a significant environmental association. SNP order on the X-axis is consistent with the Larroque et al. (2019) draft genome scaffold order.


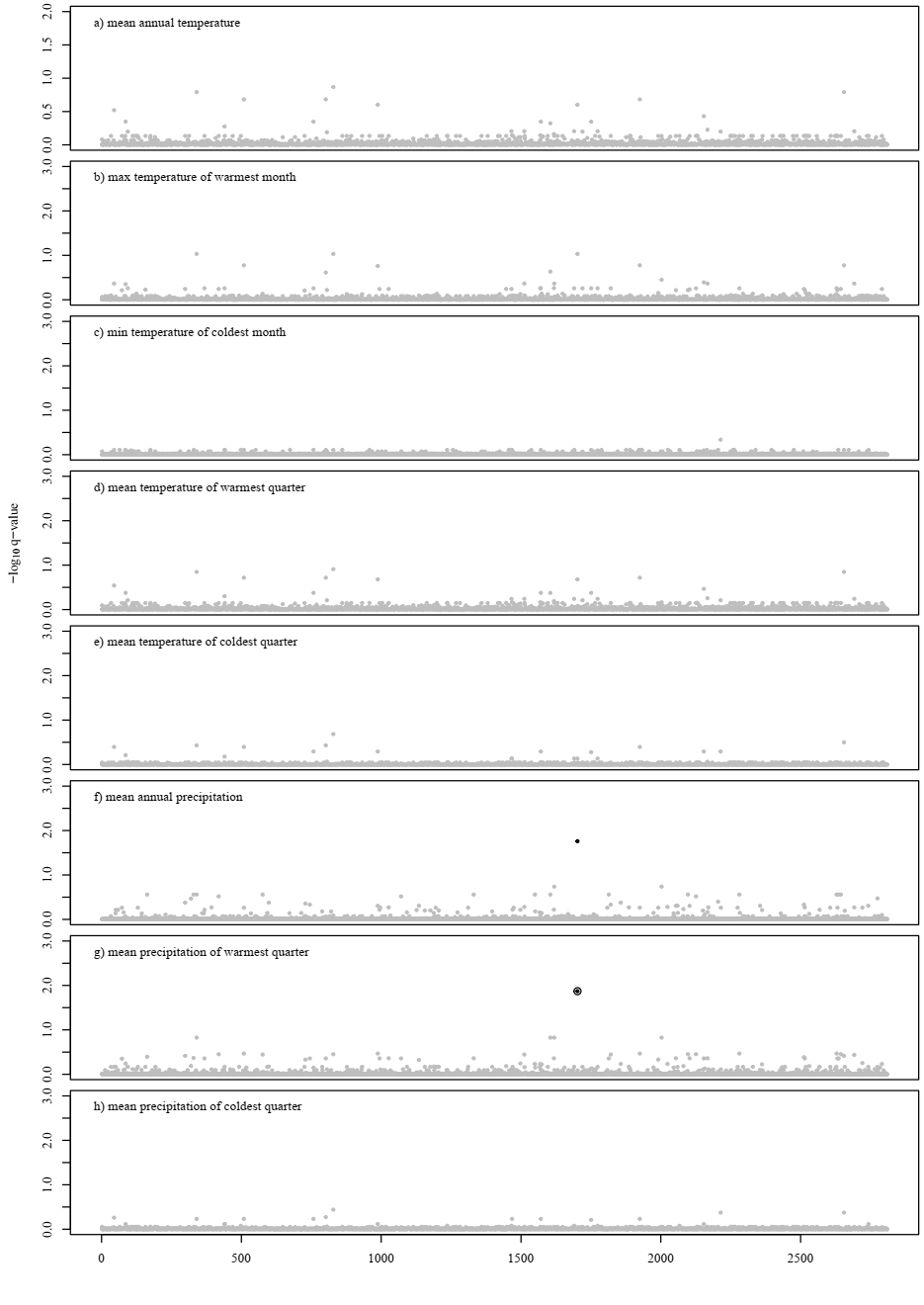


**Supplemental Figure 10.** Candidate SNPs associated with Worldclim 2 database variables derived from latent factor mixed models of *C. fumiferana* collected in Alberta. Black dots indicate loci with significant associations to the relevant environmental variable based on a *q*-value threshold of 0.05 (−log_10_ *q*-value ~1.3). Single loci often had multiple significant environmental associations due to spatial correlation of environmental variables. Open circles around black dots represent the strongest association (based on median |z|-scores) for each locus with a significant environmental association. SNP order on the X-axis is consistent with the Larroque et al. (2019) draft genome scaffold order.


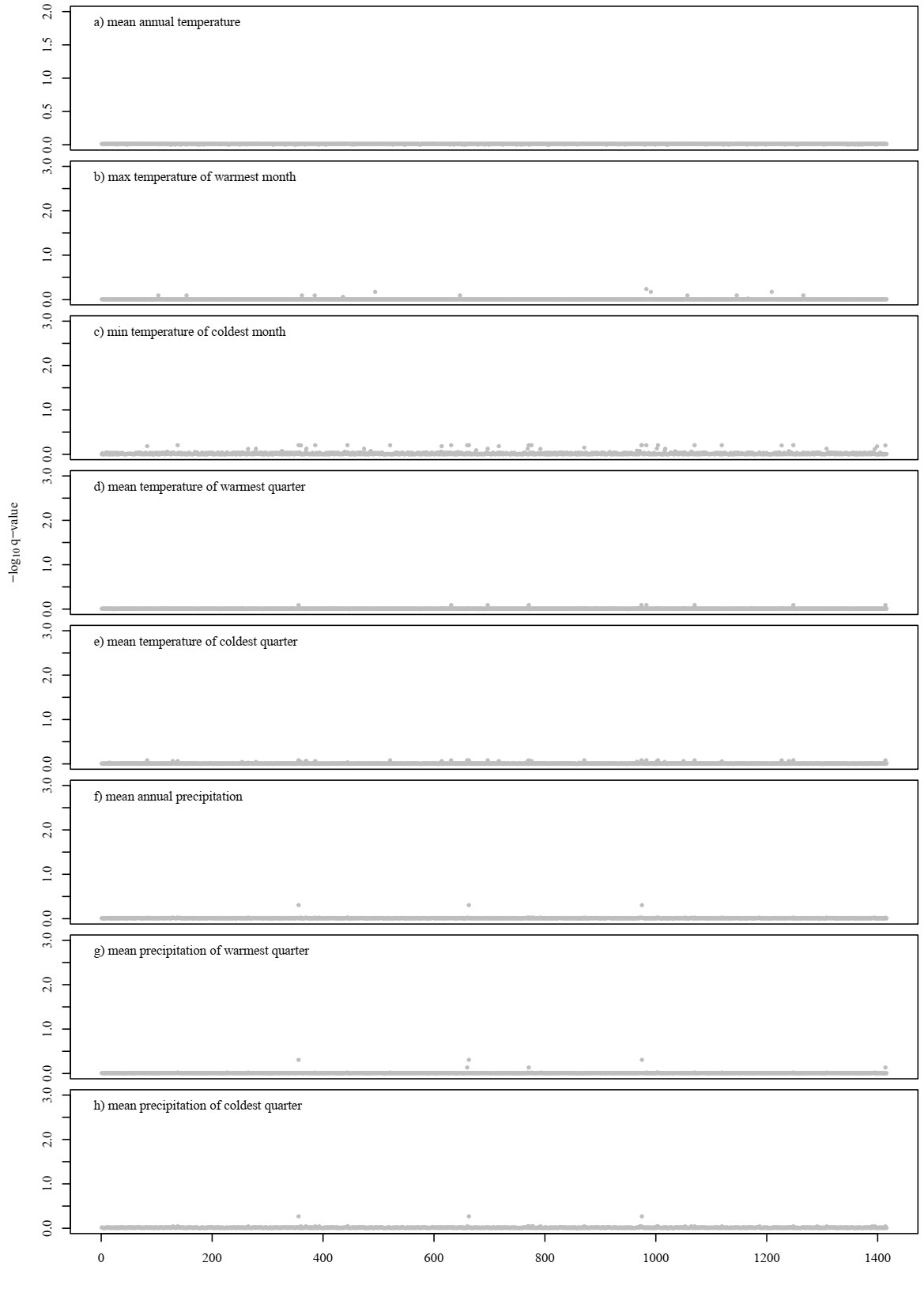


**Supplemental Figure 11.** Non-significant SNPs associated with Worldclim 2 database variables derived from latent factor mixed models of *C. o. biennis* and *C. o. occidentalis* collected in Alberta and British Columbia. SNP order on the X-axis is consistent with the Larroque et al. (2019) draft genome scaffold order.
